# Late Upper Palaeolithic hunter-gatherers in the Central Mediterranean: new archaeological and genetic data from the Late Epigravettian burial Oriente C (Favignana, Sicily)

**DOI:** 10.1016/j.quaint.2020.01.025

**Published:** 2020-01-30

**Authors:** Giulio Catalano, Domenico Lo Vetro, Pier Francesco Fabbri, Swapan Mallick, David Reich, Nadin Rohland, Luca Sineo, Iain Mathieson, Fabio Martini

**Affiliations:** 1) Dipartimento di Scienze e tecnologie biologiche, chimiche e farmaceutiche, Università degli studi di Palermo, Palermo 90128, Italy; 2) Dipartimento SAGAS – Unità di Archeologia Preistorica, Università di Firenze, Firenze 50121, Italy; 3) Museo e Istituto Fiorentino di Preistoria, Firenze 50122, Italy; 4) Dipartimento di Beni Culturali, Università del Salento, Lecce, Italy; 5) Department of Genetics, Harvard Medical School, Boston, Massachusetts 02115, USA; 6) Howard Hughes Medical Institute, Harvard Medical School, Boston, Massachusetts 02115, USA; 7) Broad Institute of MIT and Harvard, Cambridge, Massachusetts 02442, USA; 8) Department of Genetics, Perelman School of Medicine, University of Pennsylvania, Philadelphia 19104, USA

**Keywords:** Late Glacial, Late Epigravettian, funerary practices, ancient DNA, Central-Western Mediterranean, Sicily

## Abstract

Grotta d’Oriente, a small coastal cave located on the island of Favignana (Sicily, Italy) is a key site for the study of the early human colonization of Sicily. The individual known as Oriente C was found in the lower portion of an anthropogenic deposit containing typical local Late Upper Palaeolithic (Late Epigravettian) stone assemblages. Two radiocarbon dates on charcoal from the deposit containing the burial are consistent with the archaeological context and refer Oriente C to a period spanning about 14,200–13,800 cal. BP. Anatomical features are similar to those of Late Upper Palaeolithic populations of the Mediterranean and show some affinity with Palaeolithic individuals of San Teodoro (Messina, Sicily). Here we present new ancient DNA data from Oriente C. Our results, confirming previous genetic analysis, suggest a substantial genetic homogeneity among Late Epigravettian hunter-gatherer populations of Central Mediterranean, presumably as a consequence of continuous gene flow among different groups, or a range expansion following the Last Glacial Maximum (LGM).

## 1. Introduction

In the last few years, developments in sequencing techniques have enabled the generation of an unprecedented amount of genomic data from past populations. In particular, ancient genomes from Upper Palaeolithic and Mesolithic periods have made possible to explore the early genetic makeup of hunter-gatherers of the Mediterranean (Jones et al., 2015; Fu et al., 2016; Hofmanová et al., 2016; Posth et al., 2016; Modi et al., 2017; Mathieson et al., 2018). Given its geographic location and the presence of Upper Palaeolithic and Mesolithic human remains belonging to at least three individuals (Oriente A, B and C: Mannino et al., 1972; Lo Vetro and Martini, 2006; Di Salvo et al., 2012; Mannino et al., 2012; Martini et al., 2012a), Grotta d’Oriente, on the island of Favignana (NW Sicily), is a key site for the study of the human colonization of Sicily during the Upper Palaeolithic (Lo Vetro and Martini, 2012). Paleogenetic and morphological studies on the Mesolithic individual Oriente B indicated a close proximity with Late Epigravettians of the Italian peninsula (D’Amore et al., 2010; Mannino et al., 2012), while genome-wide single nucleotide polymorphism (SNP) data showed that the Upper Palaeolithic Oriente C clusters closely with other Western European Hunter-Gatherer (WHG) populations from Mesolithic and Late Palaeolithic Western Europe (Mathieson et al., 2018), confirming previous morphological analysis (Henke, 1989; Brewster et al., 2014).

Here we generated new genome-wide data in order to refine the genetic affinities of Oriente C to other European hunter-gatherer populations. Our results and additional population genetic analyses provide insights into the origin and population structure of the hunter-gatherers that inhabited Europe during the Late Upper Palaeolithic and Mesolithic.

## 2. Archaeological setting

### 2.1. The site and its setting

**Figure 1.**
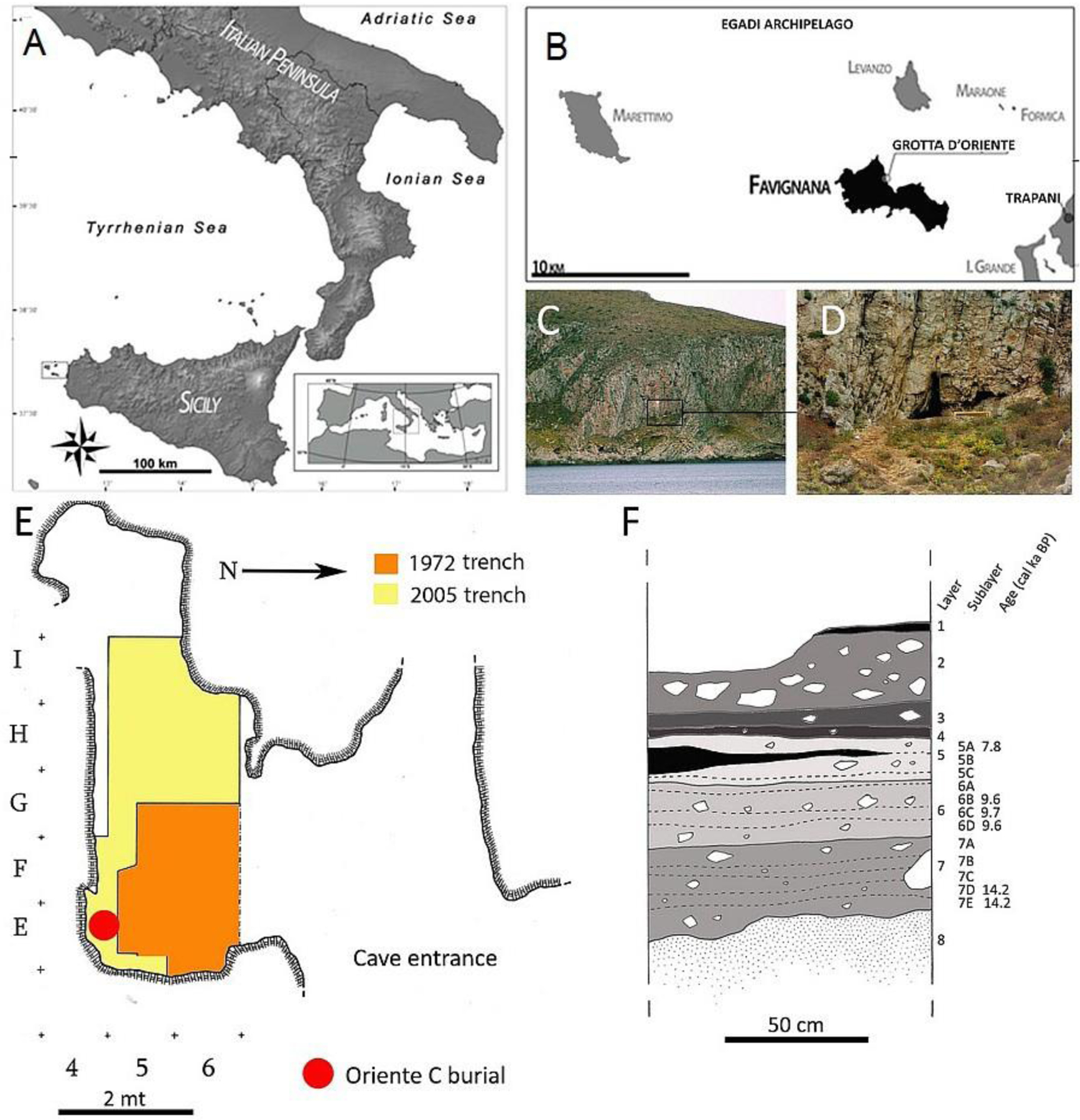
A-B) Geographic location of Grotta d’Oriente; C-D) The cave entrance on the slope of Montagna Grossa; E) Excavation areas; F) Stratigraphic sequence (2005 excavations) showing the layers and sublayers.

Grotta d’Oriente is a small coastal cave located on the island of Favignana, the largest (~20 km^2^) of a group of small islands forming the Egadi Archipelago, ~5 km from the NW coast of Sicily (Fig. 1 A–B). The cave opens on the north-eastern slope of Montagna Grossa, at ~40 m. a.s.l. (Fig. 1 C–D) and is formed of two distinct areas: a small chamber at the left of the entrance and a large gallery on the right (Martini et al., 2012b) (Fig. 1 E). Early excavations were conducted, without a strict methodology, in the small chamber in 1972 (Mannino, 1972; 2002). New excavations were performed in 2005 as a part of an interdisciplinary project carried out by the University of Florence and Museo e Istituto Fiorentino di Preistoria (Colonese et al., 2011, 2014, 2018; Craig et al., 2010; Lo Vetro and Martini, 2006; Martini et al., 2012a,b). A new trench was opened in 2005 next to the trench excavated in the 1970s and accurate recovery of materials and a microstratigraphic approach were followed. During the new excavations, a well-detailed archaeological sequence was documented (Fig. 1 F): the deposit, investigated up to a depth of about 2m, consists of 8 main sedimentological units (Layers); five of which contain evidence of human frequentation of the cave during prehistory: Late Upper Palaeolithic (Layer 7), Early Mesolithic (Layer 6), Late Mesolithic or Early Neolithic (Layer 5) and Bronze Age (Layers 4–3). These cultural deposits were further divided into sublayers each corresponding to different paleosurfaces which are often characterized by hearths and pits, and abundant artefacts and faunal remains (both terrestrial and marine) (Martini et al., 2012b; Colonese et al., 2018). These sublayers (AMS radiocarbon data are reported in Table 1) are attributable to short-term episodes of human frequentation.

At the top of the deposit there are two historical levels (Layers 1–2), with scarce pottery remains, which have been largely reworked. The lowermost level (Layer 8) contains Pleistocene fauna with no evidence of human activity.

In addition to the Palaeolithic burial Oriente C, which is the object of this study and was brought to light during the excavations in 2005 (Lo Vetro and Martini, 2006; Martini et al., 2012a), two other burials have been unearthed during the 1972 excavation campaign: Oriente A and Oriente B (Mannino, 2002). Oriente A is a Mesolithic or, most likely, a Late Palaeolithic adult male (Lo Vetro and Martini 2006, for considerations about the chronological attribution of this burial); Oriente B is a Mesolithic adult female which has recently been directly dated (three AMS radiocarbon dates, all falling within the first half of the 10th millennium uncal. BP, Mannino et al., 2012). Other human bones recovered during 1972 excavations outside of the burial contexts (Mannino et al., 2012), have been recently identified at the Museo Archeologico Regionale “Antonio Salinas” (Palermo).

### 2.2. The Oriente C burial

The Oriente C funereal pit opens in the lower portion of Layer 7, specifically sublayer 7D. Two radiocarbon dates on charcoal from the sublayers 7D (12149±65 uncal. BP) and 7E, (12132±80 uncal. BP) are consistent with the associated Late Epigravettian lithic assemblages (see Table 1) (Lo Vetro and Martini, 2012; Martini et al., 2012b) and refer the burial to a period between about 14,200–13,800 cal. BP, when Favignana was connected to the main island (Agnesi et al., 1993; Antonioli et al., 2002; Mannino et al., 2014). Several attempts to directly date Oriente C remains were performed by CEDAD laboratory (University of Salento) but all were unfortunately unsuccessful due to the lack of collagen preservation.

**Figure 2.**
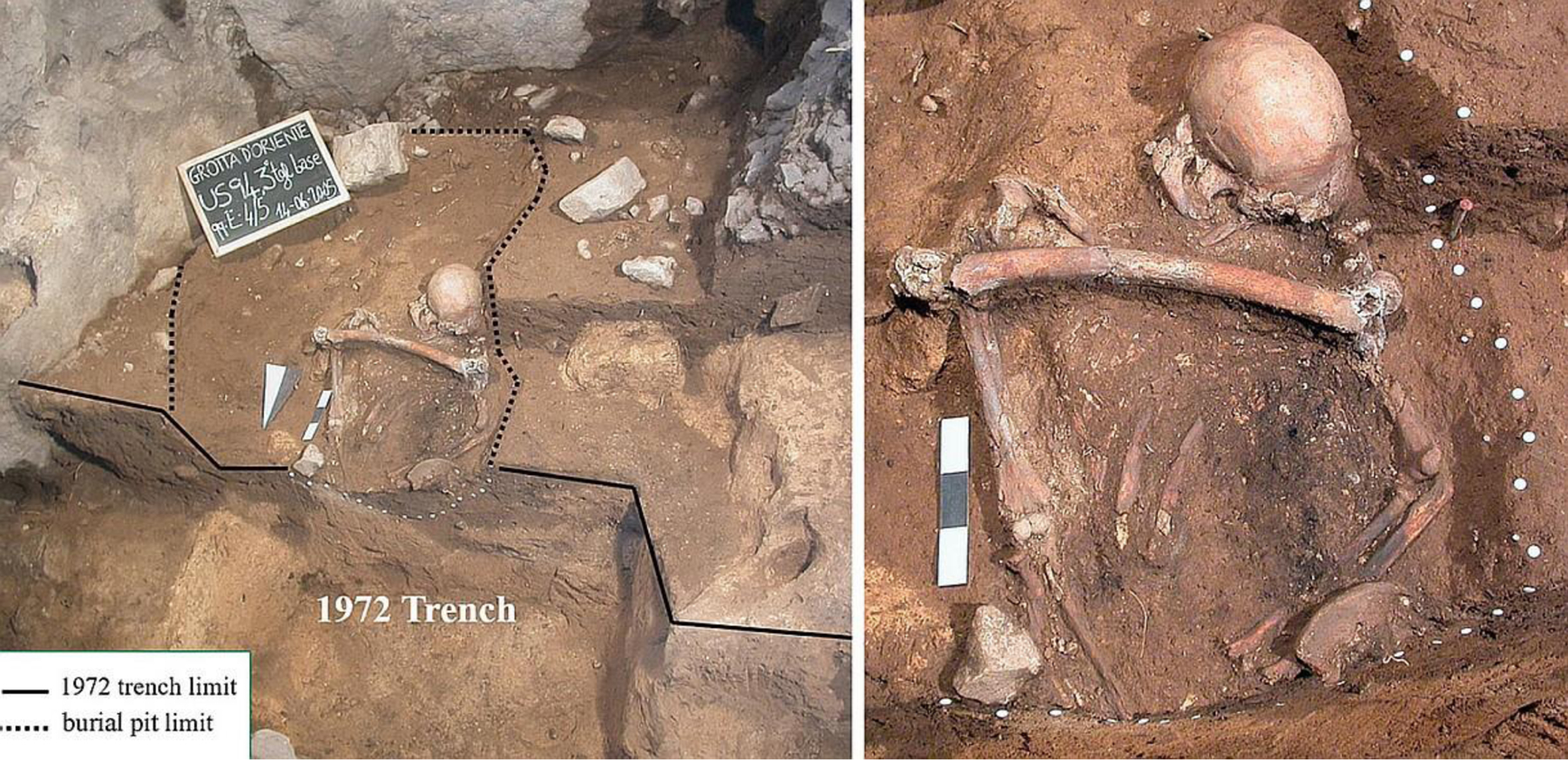
The burial Oriente C during excavations: a wide view of the burial on the left, a close-up of the skeleton on the right (Photo D. Lo Vetro).

The burial is located at the SW corner of the small chamber at the cave entrance, close to the rock wall. The skeleton is completely devoid of the lower limbs, large part of the pelvis and the hands. Based on the stratigraphic evidence, the lack of the lower portion of the skeleton could be possibly explained considering two events: 1) a small pit originated at the top of the Layer 7, during the Early Mesolithic, has partially affected the burial, perhaps removing the lower left skeletal elements; 2) the trench excavated by G. Mannino in 1972, intercepted the remaining part of skeleton at the level of the pelvis as is clearly visible in Fig. 2.

A third event could have occurred without leaving clear traces in the stratigraphic succession: a very peculiar feature of this burial is, in fact, the presence of a femur (left) placed on the thorax between the shoulders. The taphonomic study did not detect any evident post-depositional disturbance of the skeleton that could have occurred in case of a reopening of the grave; the dislocations of some bones could therefore be attributed to post-burial movements inside the funerary pit. For this reason it is likely that femur could have been deposited during the interment and thus it could belonged to another individual (perhaps a relic?). However, since femur is compatible with the remaining part of the articulated skeleton (see below), it cannot be excluded the possibility that it belongs to Oriente C. In this case, we should assume a reopening of the grave (a violation?) during the Palaeolithic, after the decomposition of the body (no cut-marks or other traces linked to defleshing are evident on the femur), and the subsequent voluntary placement on the thorax of Oriente C of his own left femur. The reopening, which has no left clear traces on the rest of the skeleton, may have caused also a dislocation of the whole lower portion of the body. If this event happened, it would have preceded the disturbance of the grave occurred during the Mesolithic (the small pit opened at the top of Layer 7A) as the grave was closed again after the deposition of the femur on the individual’s chest and sealed by the Palaeolithic deposit (Layer 7C). This hypothesis could also explain why G. Mannino did not notice the presence of Oriente C burial, which he would have to intercept with his excavation trench, as the skeleton was already devoid of the lower limbs (Mannino, 2002; Lo Vetro and Martini, 2006).

Beyond many disturbance episodes, the state of preservation of the human remains was generally very poor. For this reason, to allow the recovery of human remains avoiding irreparable damages, it was considered necessary to consolidate most of them several times with acrylic resin Paraloid B72 (ethyl-methacrylate copolymer) mixed with acetone. Subsequently, the remains were removed and restored by gluing the parts after careful cleaning of the surfaces with acetone in order to remove the Paraloid film. Before any consolidation and restoration, during the burial excavation, numerous bone fragments were examined by morphological analysis in order to separate human bones from the animal ones which were accidentally in the pit filling, as usually happens in burials excavated in multi-layered deposits.

Oriente C laid in dorsal decubitus oriented from South (the skull’s position) to North. The head rested on a large limestone chip with the face turned slightly to the left. The right upper limb was extended on the side of the trunk, while the left one was flexed (about 120°) with its lower end placed on the lower abdomen. All the bones, stored at the Laboratory of Prehistory-University of Florence (Table 2), show the same colour (red-brownish) and degree of fossilization except left ulna which is darker and left radius which is nearly black because of a small fire lit in the grave (see above). The skull, mandible and left elbow were slightly displaced, upper right limb long bones were articulated, and left iliac blade partially covered the flexed left forearm. These bones certainly belong to a single intentionally buried individual. A left femur was laid transversely above the upper part of the articulated skeleton, with the upper epiphysis on the left humerus (Fig. 2). No cut-marks or other traces linked to defleshing are evident on the femur. It is possible but not proven that the femur belongs to the same individual represented by the articulated bones (Lo Vetro and Martini, 2006).

Many other human bones, often fragmentary, were also recovered during 1972 excavations. Approximately forty anatomical elements were recently identified among the material stored at the Museo Archeologico Regionale “Antonino Salinas” in Palermo (Mannino et al., 2012). Some of these, especially the hand bones, could belong to Oriente C but currently it is not possible to establish it. Moreover, a distal left humerus and radius, and a distal right ulna also occur. Mannino et al. (2012) excluded the attribution of these limb bones to both B and C individuals, but they could not ascertain the association with Oriente A or with a fourth individual. For this reason, they attributed these bones to Oriente X. In our opinion the distal left humerus does not belong to Oriente C, because a left humerus is present in the Florence collection. Conversely, the distal left radius and right ulna, absent in Florence, could belong to Oriente C but this is a hypothesis that should be confirmed after a direct observation of the remains and a comparative analysis.

Age at death could not be accurately determined because of the lack of suitable anatomical parts. Nevertheless, we observe that exocranial sutures are not fused and there is a beginning of fusion on the endocranial aspect of the obelic suture; the lt. M3 and the fragment of upper molar (an M1 or M2) are unworn, the six preserved long bone epiphysis (inferior right and left humerus; upper right and left radius; upper and lower left ulna) are completely fused to the diaphysis and do not show traces of osteoarthritis. Hence, we conclude that the individual probably was a young adult, maybe 25–30 years old. Oriente C lacks the diagnostic parts of the hip bones, but the long bone midshaft and epiphysis measurements – commonly used in sex determination of fragmentary human remains –indicated that the individual was most likely female (see Table S1 and Figure S1 in Supplementary material). This determination was later confirmed genetically (Mathieson et al., 2018). Stratigraphic and taphonomic features suggest that the funerary ritual of Oriente C consisted of a sequence of steps that can be summarized as follows:

*1- Excavation of the funerary pit*. The pit originates in the lower part of the Layer 7 (sub-layer 7D) and affects the base of the Layer 7 (sub-layer 7E) and the underlying Layer 8 (sterile yellowish sands); it is shallow (about 25 cm) and has a flat bottom. The original mouth of the pit may have been obliterated in the case of a subsequent reopening of the pit (see step 5). The Northern portion of the pit was removed during 1972 excavations.
*2- Deposition of the body*. The individual was placed into the grave, his skull rests almost on the western edge of the pit.
*3a- Burning action 1*. After the deposition, when the soft tissues of the body were probably still present, a low-heat fire was lit at the bottom of the grave, in direct contact with the body, in the area of the lower left hemithorax. The short and weak combustion left traces on the left forearm and deposited charcoal, ashy soil, at the bottom of the pit. Following the hypothesis that femur was placed on the shoulders of the deceased during the interment, it must have occurred after the fire extinction, since femur lays on a thin layer of soil covering the charcoal and there are no traces of burning on it.
*3b- Burning action 2*. A second low-heat fire was lit to the right of the skull.
*4*- *Closing of the grave*. The individual was definitively interred.
*5-Possible reopening of the grave and deposition of a femur (if the femur belonged to Oriente C individual)*. A femur was placed between the shoulders of the body after the reopening of the grave. In this case the original mouth of the grave may have been partially obliterated and pit limits detected during the excavation may refer to the reopening of the burial. The reopening, if there was any, took place during the Late Upper Palaeolithic (top of the Layer 7D), since Layer 7C, which covers the mouth of the pit, still refers to the Late Epigravettian.
*6-Deposition of stones*. Along the eastern edge of the grave, and also inside it, limestone blocks were deposited. Some of these blocks protruding from the pit were probably placed as a marker for the identification of the location of the burial.

The available anatomical features of Oriente C are similar to those of Late Upper Palaeolithic populations of the Mediterranean and show affinity with other Palaeolithic individuals of Sicily, but the fragmentary cranial remains of Oriente C do not permit a deeper morphological analysis. As suggested by Henke (1989) and Fabbri (1995) the hunter-gatherer populations were morphologically rather uniform. This interpretation is further supported by the low or negligible *D*^*2*^ distance demonstrated by D’Amore et al. (2009) in the comparison between San Teodoro (individuals 1–2-3–5-7) craniofacial morphometrics and other Upper Palaeolithic individuals. Like other Late Epigravettian burials in Sicily and Italy (Palma di Cesnola, 2003; Giacobini, 2007), Oriente C is a simple burial with little or no grave goods and personal ornaments. The only items in the pit were a pierced shell of *Cerithium* sp. (perhaps a clothing ornament) and a few small lumps of red ochre, next to the skull and the femoral head. Stable isotope analysis suggested a largely terrestrial diet with low-level consumption of marine foods which is comparable to other Late Upper Palaeolithic individuals from Sicily and Italy (Craig et al., 2010; Mannino et al., 2012).

## 3. Genetic analysis

A first attempt to analyse mitochondrial DNA from a rib fragment of Oriente C, performed in 2006 by University of Rome “Tor Vergata” (Lo Vetro and Martini, 2006), was unsuccessful likely because made on a fragment too compromised by restoration procedures. The current ancient DNA analysis was done on a single long bone fragment that was not exposed to the substances used for consolidation and restoration (see above section 2.2). Sample preparation, DNA extraction and library construction were carried out in dedicated ancient DNA facilities in Boston as described in (Mathieson et al., 2018). To increase coverage compared to the previously reported data, we generated two additional libraries from the same extract, performed in-solution enrichment (“1240k”) and sequenced the product on an Illumina NextSeq500 using v.2 150 cycle kits for 2 × 76 cycles and 2 × 7 cycles. We merged these data with the data from the original library and made pseudo-haploid calls by selecting a single sequence at each single nucleotide polymorphism (SNP). The resulting dataset contained information on 288,223 SNPs covered at least once, compared to 61,547 in a previous publication (Mathieson et al., 2018), allowing for higher resolution analysis. To investigate the genetic affinities of Oriente C, we used the *qp3Pop* program from ADMIXTOOLS (Patterson et al., 2012) to compute *f*_*3*_-statistics and to estimate the amount of shared genetic drift between Oriente C and 98 published Mesolithic and Late Palaeolithic hunter-gatherers (with coverage at a minimum of 20,000 of 1240k positions) from 30 sites across Europe (Gamba et al., 2014; Gonzales-Fortes et al., 2017; Gunther et al., 2018; Haak et al., 2015; Jones et al., 2015; Lazaridis et al., 2014; Lipson et al., 2017; Mathieson et al., 2015, 2018; Olalde et al., 2014). We used the *qpDstat* program to estimates *D*-statistics to test whether pairs of populations form a clade. The statistic *D* (*outgroup, A, B, C*) is zero if A is an outgroup to the clade (B, C), positive if A is closer to C, and negative if it is closer to B. For *D*- and *f*_*3*_-statistics, we estimated standard errors using the default block jackknife procedure implemented in ADMIXTOOLS (Patterson et al., 2012).

We confirmed the originally reported mitochondrial haplogroup assignment of U2’3’4’7’8’9. This haplogroup is present in both pre- and post-LGM populations, but is rare by the Mesolithic, when U5 dominates (Posth et al., 2016). We further confirmed that the new genome-wide data was consistent with the original data by computing *D*-statistics (Patterson et al., 2012) of the form *D (Mbuti, X, Original Oriente data, Merged Oriente data)*. None of these statistics were significantly non-zero when X ranged over other European Mesolithic hunter-gatherers (maximum |Z| = 1.8 in 34 tests), and present-day French (Z = −0.35) and Sardinian (Z = −0.13) populations.

**Figure 3.**
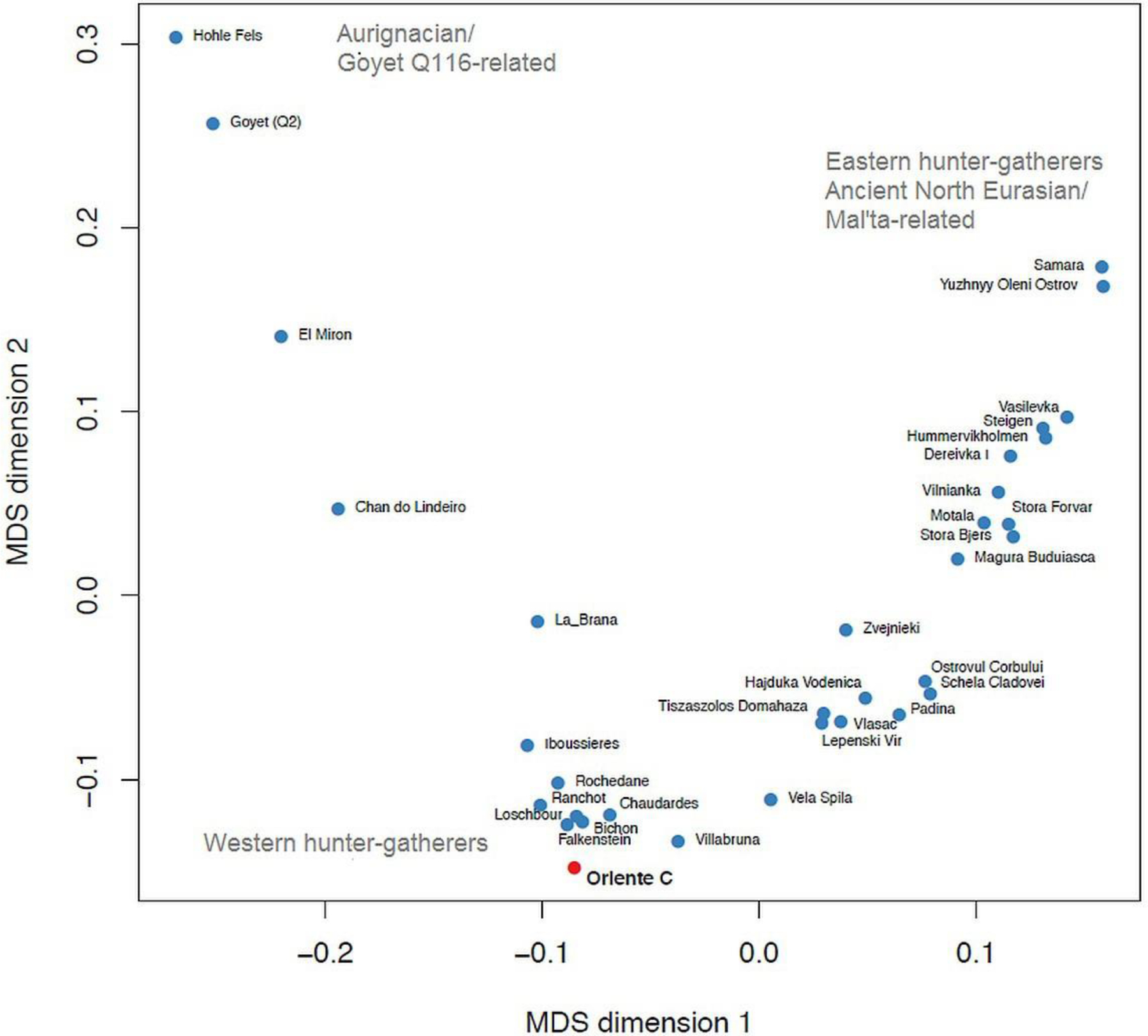
Multidimensional scaling of outgroup *f*_*3*_-statistics for Late Upper Palaeolithic and Mesolithic hunter-gatherers.

Lipson et al. (2018) (their supplementary Figure S5.1) and Villalba-Mouco et al. (2019) (their Figure 2A) showed that European Late Palaeolithic and Mesolithic hunter-gatherers fall along two main axes of genetic variation. Multidimensional scaling (MDS) of *f*_*3*_-statistics shows that these axes form a “V” shape (Fig. 3). At the root of the “V” lie the individuals that have been described as belonging to the “Western hunter-gatherer” (WHG) population, clustering closely with the 8,000 BP Loschbour individual (Lazaridis et al., 2014). One arm represents a cline of ancestry that links WHG with “Eastern hunter-gatherer” (EHG) populations who carry ancestry related to the “Ancient North Eurasian” (ANE) population represented by the 24,000 BP Mal’ta individual (Raghavan et al., 2014). Along this cline lie Eastern European hunter-gatherer populations such as those from the Balkan Peninsula, present-day Ukraine (Mathieson et al., 2018), the Baltic (Jones et al., 2017; Mathieson et al., 2018) and Scandinavia (Haak et al., 2015, Gunther et al., 2018). The other arm of the “V” is a cline containing Late Upper Palaeolithic and Mesolithic individuals from Iberia (for example the individuals from El Mirón and La Braña), and Late Upper Palaeolithic individuals from Central Europe (Goyet and Hohle Fels). As shown by Fu et al. (2016), this cline reflects an ancestry contribution from a population related to the 35,000 BP Aurignacian Goyet Q116–1 individual. In this analysis, Oriente C falls at the tip of the “V”, at the extreme end of the WHG grouping.

**Figure 4.**
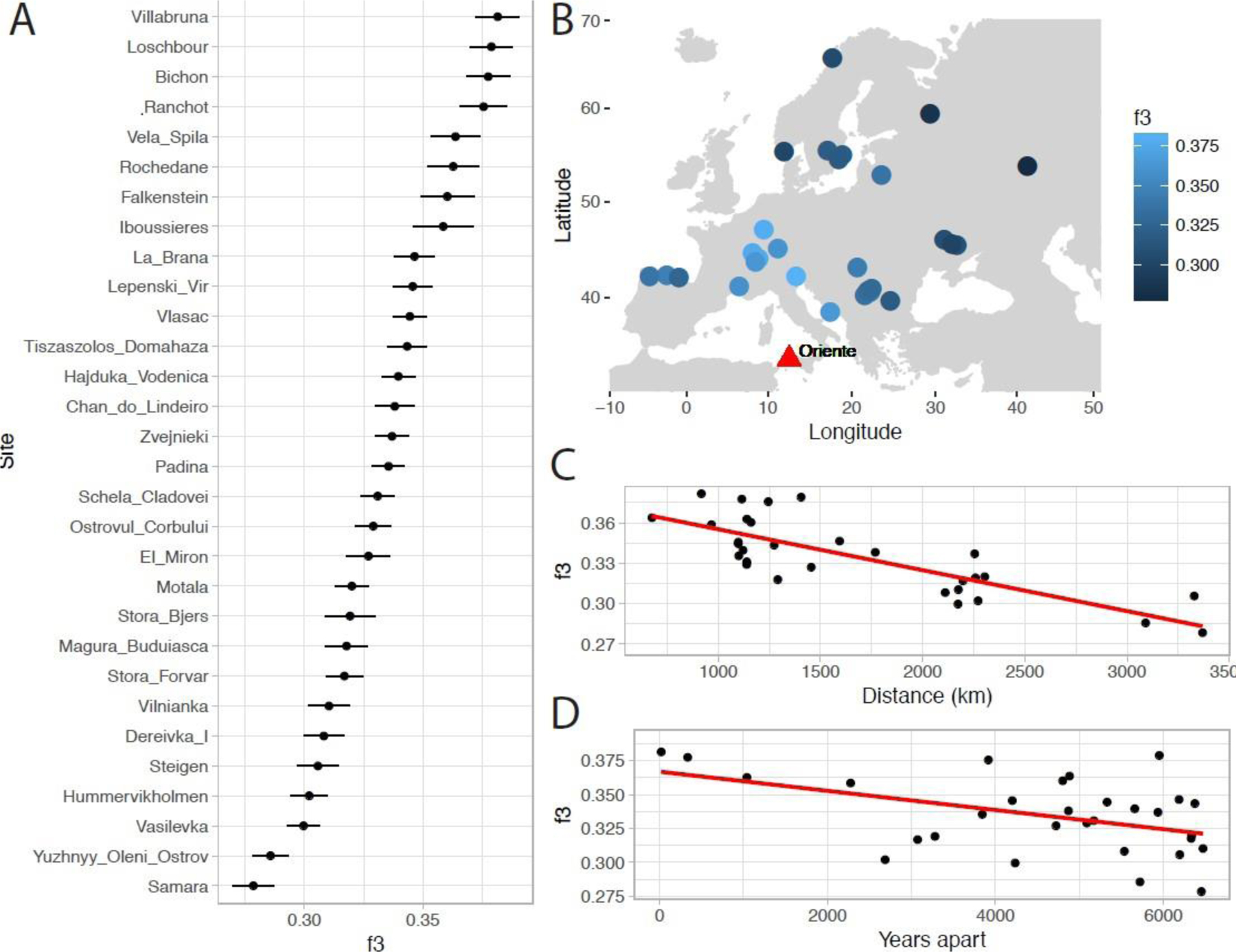
A) Shared drift, estimated using *f*_*3*_-statistics between Oriente C and 98 Mesolithic and Late Palaeolithic hunter-gatherers from 30 sites; B) The same statistic as in A plotted with geographic position; C) Decay of shared drift with distance from Oriente C; D) Decay of shared drift with absolute difference in date from Oriente C.

Focusing further on Oriente C, we find that it shares most drift with individuals from Northern Italy, Switzerland and Luxembourg, and less with individuals from Iberia, Scandinavia, and East and Southeast Europe (Fig. 4A–B). Shared drift decreases significantly with distance (Fig. 4C) and with time (Fig. 4D) although in a linear model of drift with distance and time as a covariate, only distance (p=1.3×10^−6^) and not time (p=0.11) is significant. Consistent with the overall E-W cline in hunter-gatherer ancestry, genetic distance to Oriente C increases more rapidly with longitude than latitude, although this may also be affected by geographic features. For example, Oriente C shares significantly more drift with the 8,000 year-old 1,400 km distant individual from Loschbour in Luxembourg (Lazaridis et al., 2014), than with the 9,000 year old individual from Vela Spila in Croatia (Mathieson et al., 2018) only 700 km away as shown by the D-statistic (Patterson et al., 2012) D (Mbuti, Oriente C, Vela Spila, Villabruna); Z=3.42. Oriente C’s heterozygosity was slightly lower than Villabruna (14% lower at 1240k transversion sites), but this difference is not significant (bootstrap P=0.12). The low coverage of the sample meant that its genotype at any particular site–for example those directly related to phenotypic traits–could not be reliably ascertained. However, at sites with coverage, Oriente C appears consistent with other Western Hunter Gatherers. For example, it carries the ancestral allele (2 reads) at the *SLC45A2* pigmentation-associated SNP rs16891982 and (3 reads) at the lipid metabolism-associated SNP rs174546 in the *FADS* gene cluster. Oriente C’s genotype at other phenotypically associated sites including the *LCT* lactase persistence SNP and the immune-associated *TLR* cluster could not be determined, however.

## 4. Discussion

Sicily falls within the area of expansion of the Epigravettian model widespread after the LGM in Mediterranean Europe, from Provence to the eastern Balkan border up to the Black Sea and the SW Anatolia. This “cultural province” is characterized by peculiar features, rooted in the local Gravettian traditions, which concern not only lithotechnics but also artistic production and burial customs (Kozlowski, 2005 and reference therein; Fontana et al., in press). The Epigravettian (about 21.0–11.5 cal. ka BP) is a quite homogeneous cultural phenomenon despite the paleo-environmental differences occurring in a wide territory and some differentiations in resources exploitation strategies and human-environment interactions which were, perhaps, responsible for the formation of regional variants (Palma di Cesnola, 1993; Kozlowski, 2005). In Italy a regionalization of Epigravettian stone assemblages is more clearly recognizable at the end of the Late Glacial (on this topic see Palma di Cesnola, 1993 and the several contributions in Martini, 2007). Recently the regionalist hypothesis has been criticized by Tomasso (2017) in a paper where the evidence from the southernmost part of the Italian peninsula and Sicily was not considered.

In Sicily, where the only reliable evidence belongs to the Late Epigravettian (about 16–11.500 cal. BP), this culture presents very specific local features, especially in lithic assemblages (Martini et al., 2007; Lo Vetro and Martini, 2012). The figurative production is the element that more than others seems to connect Sicily to the peninsular Epigravettian sphere; the iconographic languages of Sicilian Late Epigravettian fit very well in the so-called “Mediterranean style” *sensu* Graziosi (1956). Although this definition is no longer appropriate to the current archaeological context (Vigliardi, 1996, 2005; Bicho et al., 2007; Martini, 2016), it highlights the existence of stylistic affinities in the Upper Palaeolithic art of some areas of Central-Western Mediterranean Europe. A further element that connects Sicily to the Italian peninsula and more generally to the Western Europe consists in the so-called “Azilian” artistic productions (i.e. painted pebbles from Grotta di Cala del Genovese in Levanzo island), a phenomenon that seems to have crossed the Tyrrhenian side of the Italian peninsula from Liguria to the southern regions (Martini, 2016 and reference therein). Even the funeral practices (i.e. Grotta di San Teodoro) show strong affinity with the peninsular evidence (Palma di Cesnola, 2003; Giacobini, 2007), from Calabria (i.e. Grotta del Romito) (Martini and Lo Vetro, 2018 and reference therein) up to Veneto (i.e. Riparo Tagliente and Riparo Villabruna) (Broglio, 1995; Bartolomei et al., 1974). Industrial and symbolic evidence, therefore, suggest that during the Late Glacial period Sicily was fully included in the “Epigravettian cultural province” and for this reason we can consider Sicily as the most continental of the Mediterranean islands.

Numerous archaeological sites related to the human frequentation of Sicily in the Late Upper Palaeolithic reveal that Late Epigravettian hunter-gatherer groups inhabited intensively the island during the Late Glacial period (Lo Vetro and Martini, 2012). The considerable record of radiocarbon dates proves that they reached Sicily not before 16–14 ka cal. BP, several millennia after the LGM peak, during a period of sudden sea level rise which caused a dramatic transformation of the coastal morphology of the island (Lambeck et al., 2011; Lo Presti et al., 2019). In our opinion, in fact, the hypothesis about an early colonization of Sicily by Aurignacians (Laplace, 1964; Chilardi et al., 1996) must be rejected on the basis of a recent re-interpretation of the techno-typological features of the lithic assemblage from Riparo di Fontana Nuova which has been re-assigned to the Late Epigravettian (Martini et al., 2007; Lo Vetro and Martini, 2012; on this topic see also Di Maida et al., 2019).

The Late Upper Palaeolithic Oriente C is a simple burial, and its sober ritual and the modality of deposition fit very well in the context of the Late Epigravettian burials of Sicily and Central-Southern Italy (Palma di Cesnola, 2003; Giacobini, 2007). Regarding the funerary ritual, an interesting issue, unique in the panorama of the Palaeolithic burials in Europe, concerns the peculiar occurrence of a femur placed on the shoulders of the individual and its possible belonging to the skeleton found in place.

Many sources of evidence indicate that the LGM may have had a major role in shaping the genetic and phenotypic variation of Upper Palaeolithic populations. A recent study based on complete mitochondrial genomes has revealed a genetic homogeneity between European hunter-gatherers. A significative predominance of the U lineage was detected with most of the sequences belonging to U5 haplotypes (Posth et al., 2016). The finding of the haplogroup U2’3’4’7’8’9 in the Oriente C individual, previously recovered in the Upper Palaeolithic humans from Grotta Paglicci (Posth et al., 2016) provides additional evidence for the hypothesis that Epigravettian culture might have reached Sicily during the migration of Upper Palaeolithic groups from Southern Italy after the LGM (Palma di Cesnola, 2006; Lo Vetro and Martini, 2012; Mannino et al., 2012), which accords with the morphological similarity of Late Upper Palaeolithic and Early Mesolithic populations in the region (Henke, 1989; Brewster et al., 2014). The find of genetic similarity of Oriente C with Late Upper Palaeolithic and Mesolithic individuals from Northern Italy (i.e. Villabruna) and Central Europe (i.e. Bichon, Loschbour) (Fig. 3) is also in line with previous studies according to which Sicilian hunter-gatherers were found to be morphologically closely related to Late Epigravettian individuals of the Italian peninsula and continental Europe (Fabbri, 1995; D’Amore et al., 2010).

## 5. Conclusions

These analyses have implications for the understanding of the origin and diffusion of the hunter-gatherers that inhabited Europe during the Late Upper Palaeolithic and Mesolithic. Our findings indicate that Oriente C shows a strong genetic relationship with Central and Western European Late Upper Palaeolithic and Mesolithic hunter-gatherers, suggesting that the “Western hunter-gatherers” was a genetically homogeneous population widely distributed in the Central Mediterranean. In our opinion this geographic structure may reflect a continuous gene flow among different groups or at least partly may represent the legacy of South to North migration events after the Late Glacial period which replaced almost all of the pre-LGM ancestry in Central and Western Europe. At the same time, it is risky to force a correlation between evidences that work on different resolution scales. The archaeological record available for the final Epigravettian of Southern Italy is considerable in terms of absolute chronology. On the other hand, it is not possible to elaborate reliable hypotheses based on genetic data only, given the paucity of Epigravettian genomes analyzed so far. Hence, we believe that more paleogenomic data for the chrono-cultural segment spanning between 20 to 16 ka BP (ancient and evolved Epigravettian) will allow a more comprehensive understanding of the dynamics of European hunter-gatherer populations in the period immediately after the LGM.

In conclusion, the DNA study of Oriente C is particularly relevant to improve the knowledge about the peopling of the Central Mediterranean by Anatomically Modern Humans after the LGM. The data support the hypothesis that hunter-gatherer groups arrived in Sicily from the Italian peninsula, confirming results derived from anatomical studies on human fossil remains of Grotta di San Teodoro and from material productions (lithic and figurative) whose characteristics fall within the Late Epigravettian physiognomy of Southern Italy, albeit with some peculiar features, especially in lithic productions, which reveal a regional identity.

## Supplementary Material

1

## Figures and Tables

**Table 1.** Grotta d’Oriente, radiocarbon dates on charcoals from the stratigraphic sequence (2005 excavations). ^14^C ages are reported as conventional and calibrated years BP, the dates were calibrated with OxCal v4.3 using the IntCal13 calibration curve (Reimer et al., 2013). The dates were performed at the CEDAD (Centre for Dating and Diagnostics), University of Salento, Italy.

Lab. Code	Layer	14C yr BP	14C yr cal. BP (68%)	14C yr cal. BP (95%)	Cultural period	Reference
LTL877A	5A	7040 ± 55	7940 – 7829	7969 – 7741	Late Meso-Early Neolithic	Martini et al., 2012b
LTL876A	6B	8619 ± 65	9660 – 9530	9762 – 9485	Early Mesolithic	Martini et al., 2012b
LTL874A	6C	8608 ± 65	9658 – 9526	9737 – 9480	Early Mesolithic	Martini et al., 2012b
LTL875A	6D	8699 ± 60	9732 – 9551	9888 – 9542	Early Mesolithic	Martini et al., 2012b
LTL14260A	7D	12149 ± 65	14136 – 13932	14195 – 13791	Upper Palaeolithic	Unpublished
LTL873A	7E	12132 ± 80	14107 – 13853	14198 – 13765	Upper Palaeolithic	Lo Vetro and Martini, 2006; Martini et al., 2012b

**Table 2.** Oriente C skeletal remains housed at the Museo e Istituto Fiorentino di Preistoria in Florence.

Anatomical region	Bone	Preservation
Skull	cap	with fragmentary base
	mandibula	fragments of mandibular rami
	teeth	-left M^3^ -fragment of an upper molar larger than M^3^ -dental fragments
Upper limbs	right humerus	-fragmentary lower third diaphysis -fragments of lower epiphysis
	left humerus	lower epiphysis
	right radius	head
	left radius	-upper epiphysis -fragments of diaphysis
	right ulna	upper 2/3 of diaphysis
	left ulna	-upper and lower epiphysis missing styloid process -fragmentary diaphysis
Lower limbs	left iliac bone	fragment comprising the anterior superior Iliac spine
	left femur	missing lower epiphysis
	left 3^rd^ metatarsal	-

## Data Availability

The 1240k capture sequencing data for Oriente C (merged new and existing data) has been deposited in the European Nucleotide Archive (https://www.ebi.ac.uk/ena) under accession number PRJEB33231.

## References

[R1] Agnesi V, Macaluso T, Orrù P, Ulzega A, 1993. Paleogeografia dell’Arcipelago delle Egadi (Sicilia) nel Pleistocene superiore-Olocene, Naturalista siciliano, S. IV, XVII (1–2), pp. 3–22.

[R2] Antonioli F, Cremona G, Immordino F, Puglisi C, Romagnoli C, Silenzi S, Valpreda E, Verrubbi V, 2002. New data on the Holocenic sea-level rise in NW Sicily (Central Mediterranean Sea), Global and Planetary Change, pp. 121–140.

[R3] Bartolomei G, Broglio A, Guerreschi A, Leonardi P, Peretto C, Sala B, 1974. Una sepoltura epigravettiana nel deposito pleistocenico del Riparo Tagliente in Valpantena (Verona). Rivista di Scienze Preistoriche 29(1), 101–52.

[R4] Bicho N, Carvalho AF, Gonzalez-Sainz C, Sanchidrian JS, Villaverde V, Straus LG, 2007. The Upper Paleolithic Rock Art of Iberia. Journal of Arch. Met. and Th 14, 1. 10.1007/s10816-007-9025-5.

[R5] Brewster C, Meiklejohn C, von Cramon-Tauba del N, Pinhasi R, 2014. Craniometric analysis of European Upper Palaeolithic and Mesolithic samples supports discontinuity at the Last Glacial Maximum. Nat. Commun 5, 4094. 10.1038/ncomms5094. 24912847 PMC5010115

[R6] Broglio A, 1995. Les sépultures Épigravetiennes de la Vénétie (abri Tagliente et abri Villabruna). In Otte M (ed.), Actes du Colloque International de Liège, 13–17 décembre 1993. Liège: Études et Recherches Archéologiques de l’Université de Liège 68, 847–869.

[R7] Chilardi S, Frayer DW, Gioia P, Macchiarelli R, Mussi M, 1996. Fontana Nuova di Ragusa (Sicily, Italy): southernmost Aurignacian site in Europe. Antiquity 70, 553–563.

[R8] Colonese AC, Zanchetta G, Drysdale RN, Fallick AE, Manganelli G, Lo Vetro D, Martini F, Di Giuseppe Z, 2011. Stable isotope composition of late Pleistocene-Holocene Eobania vermiculata (Müller, 1774) (Pulmonata, Stylommatophora) shells from the Central Mediterranean basin: data from Grotta d’Oriente (Favignana, Sicily). Quat. Int 244, 76–87. 10.1016/j.quaint.2011.04.035.

[R9] Colonese AC, Lo Vetro D, Martini F, 2014. Holocene coastal change and intertidal mollusc exploitation in the central Mediterranean: variations in shell size and morphology at Grotta d’Oriente (Sicily). Archaeofauna 23, 181–192.

[R10] Colonese AC, Lo Vetro D, Landini W, Di Giuseppe Z, Hausmann N, Demarchi B, d’Angelo C, Leng MJ, Incarbona A, Whitwood AC, Martini F, 2018. Late Pleistocene-Holocene coastal adaptation in central Mediterranean: Snapshots from Grotta d’Oriente (NW Sicily). Quat. Int 493, 114–126. 10.1016/j.quaint.2018.06.018.

[R11] Craig OE, Biazzo M, Colonese AC, Di Giuseppe Z, Martinez-Labarga C, Lo Vetro D, Lelli R, Martini F, Rickards O, 2010. Stable isotope analysis of Late Upper Palaeolithic humans and fauna remains from Grotta del Romito (Cosenza), Italy. J. Archaeol. Sci 37, 2504–2512. 10.1016/j.jas.2010.05.010

[R12] D’Amore G, Di Marco S, Tartarelli G, Bigazzi R, Sineo L, 2009. Late Pleistocene human evolution in Sicily: Comparative morphometric analysis of Grotta di San Teodoro craniofacial remains. J. Hum. Evol 56, 537–550. 19446307 10.1016/j.jhevol.2009.02.002

[R13] D’Amore G, Di Marco S, Di Salvo R, Messina A, Sineo L, 2010. The early peopling of Sicily: evidence from the Mesolithic skeletal remains from Grotta d’Oriente. Ann. of Hum. Biol 37, 403–426. 20412025 10.3109/03014461003712947

[R14] Di Maida G, Mannino MA, Krause-Kyora B, Jensen TZT, Talamo S, 2019. Radiocarbon dating and isotope analysis on the purported Aurignacian skeletal remains from Fontana Nuova (Ragusa, Italy). PLoS ONE 14(3): e0213173. 10.1371/journal.pone.0213173 30893326 PMC6426221

[R15] Di Salvo R, Mannino G, Mannino MA, Schimmenti V, Sineo L, Thomas KD, 2012. Le sepolture della Grotta d’Oriente (Favignana). Atti della XLI Riunione Scientifica dell’Istituto Italiano di Preistoria e Protostoria: Dai Ciclopi agli Ecisti: società e territorio nella Sicilia preistorica e pro- tostorica, 341–351.

[R16] Fabbri PF, 1995. Dental anthropology of the Upper Palaeolithic sample from San Teodoro and inferences on the peopling of Italy. Zeitschriftfür Morphologie und Anthropologie 80, 311–327.

[R17] Fontana F, Lo Vetro D, Martini F, Peresani M, Ricci G, in press, L’Epigravettiano recente-finale in Italia: nuovi dati sugli aspetti locali e interregionali nel Tardoglaciale, Atti della 51° Riunione Scientifica dell’IIPP, “Italia tra Mediterraneo ed Europa: mobilità, interazioni e scambi”, Forlì 2016.

[R18] Fu Q, Posth C, Hajdinjak M, Petr M, Mallick S, Fernandes D, , 2016. The genetic history of Ice Age Europe. Nature 534, 200–205. 10.1038/nature17993. 27135931 PMC4943878

[R19] Gamba C, Jones ER, Teasdale MD, McLaughlin RL, Gonzales-Fortes G, Mattiangeli V, , 2014. Genome flux and stasis in a five millennium transect of European prehistory. Nat. Commun 5. 10.1038/ncomms6257. PMC4218962 25334030

[R20] Giacobini G, 2007. Richness and Diversity of Burial Rituals in the Upper Palaeolithic. Diogenes, 214, 19–39.

[R21] Gonzalez-Fortes G, Jones ER, Lightfoot E, Bonsall C, Lazar C, Grandal-d’Anglade A, , 2017. Paleogenomic Evidence for Multi-generational Mixing between Neolithic Farmers and Mesolithic Hunter-Gatherers in the Lower Danube Basin. Curr. Biol 27,1801–1810. 10.1016/j.cub.2017.05.023. 28552360 PMC5483232

[R22] Graziosi P, 1956. L’Arte dell’antica Età della pietra. Firenze.

[R23] Gunther T, Malmström H, Svensson EM, Omrak A, Sanchez-Quinto F, Kılınc GM, , 2018. Population genomics of Mesolithic Scandinavia: Investigating early post glacial migration routes and high-latitude adaptation. PloS Biol. 16, e2003703. 10.1371/journal.pbio.2003703. 29315301 PMC5760011

[R24] Haak W, Lazaridis I, Patterson N, Rohland N, Mallick S, Llamas B, , 2015. Massive migration from the steppe was a source for Indo-European languages in Europe. Nature 522, 207–211. 10.1038/nature14317. 25731166 PMC5048219

[R25] Henke W, 1989. Biological distances in Late Pleistocene and Early Holocene human population in Europe. In Hershkovitz I. (ed.), “People and Culture in change”, Proc. of the 2nd Symp. on Up. Paleol., Mesol. and Neol. populat. of Europe and the Medit. Bas., Tel Aviv, Sept. 6-10, 1987. BAR Int. Ser. 508, 541–563.

[R26] Hofmanová Z, Kreutzer S, Hellenthal G, Sell C, Diekmann Y, Diez-del-Molino D, , 2016. Early farmers from across Europe directly descended from Neolithic Aegeans. Proc. Natl. Acad. Sci. U.S.A 113, 6886–6891. 10.1073/pnas.1523951113. 27274049 PMC4922144

[R27] Jones ER, Gonzalez-Fortes G, Connell S, Siska V, Eriksson A, Martiniano R, , 2015. Upper Palaeolithic genomes reveal deep roots of modern Eurasians. Nat. Commun 6. 10.1038/ncomms9912. PMC4660371 26567969

[R28] Jones ER, Zarina G, Moiseyev V, Lightfoot E, Nigst PR, Manica A, , 2017. The Neolithic Transition in the Baltic Was Not Driven by Admixture with Early European Farmers. Curr. Biol 27, 576–582. 10.1016/j.cub.2016.12.060. 28162894 PMC5321670

[R29] Kozlowski JK, 2005, Paléolithique supérieur et Mésolithique en Méditerranée: cadre culturel, L’Anthropologie 109, 520–540.

[R30] Lambeck K, Antonioli F, Anzidei M, Ferranti L, Leoni G, Scicchitano G, Silenzi S, 2011. Sea level change along Italian coast during Holocene and a projection for the future. Quat. Int 232, 250–257. 10.1016/j.quaint.2010.04.026.

[R31] Laplace G, 1964, Les subdivisions du leptolithique italien. Étude de typologie analytique. Bullettino di Paletnologia Italiana 73, 25–63.

[R32] Lazaridis I, Patterson N, Mittnik A, Renaud G, Mallick S, Kirsanow K, , 2014. Ancient human genomes suggest three ancestral populations for present-day Europeans. Nature 513, 409–413. 10.1038/nature13673. 25230663 PMC4170574

[R33] Lipson M, Szécsényi-Nagy A, Mallick S, Pósa A, Stégmár B, Keerl V, , 2017. Parallel palaeogenomic transects reveal complex genetic history of early European farmers. Nature 551, 368–372. 10.1038/nature24476. 29144465 PMC5973800

[R34] Lipson M, Cheronet O, Mallick S, Rohland N, Oxenham M, Pietrusewsky M, , 2018. Ancient genomes document multiple waves of migration in Southeast Asian prehistory. Science 361, 92–95. 10.1126/science.aat3188. 29773666 PMC6476732

[R35] Lo Presti V, Antonioli F, Palombo MR, Agnesi V, Biolchi S, Calcagnile L, Di Patti C, Donati S, Furlani S, Merizzi J, Pepe F, Quarta G, Renda P, Sulli A, Tusa S, 2019. Palaeogeo-graphical evolution of the Egadi Islands (western Sicily, Italy). Implications for late Pleistocene and early Holocene sea crossings by humans and other mammals in the western Mediterranean. Earth-Sci. Rev 194, 160–181.

[R36] Lo Vetro D, Martini F, 2006. La nuova sepoltura epigravettiana di Grotta d’Oriente (Favignana, Trapani). In Martini F (ed.), La cultura del Morire nelle società preistoriche e protostoriche italiane. Studio interdisciplinare dei dati e loro trattamento informatico. Origines, Progetti, vol. 1. Istituto Italiano di Preistoria e Protostoria, Firenze, 58–66.

[R37] Lo Vetro D, Martini F, 2012. Il Paleolitico e il Mesolitico in Sicilia. In: Atti XLI Riunione Scientifica IIPP, “Dai Ciclopi agli Ecisti: società e territorio nella Sicilia preistorica e protostorica”. San Cipirello, Italy, 19–48.

[R38] Mannino G, 1972. Grotta d’Oriente. Rivista di Scienze Preistoriche XXVII (2): 470.

[R39] Mannino G, 2002. La Grotta d’Oriente di Favignana (Egadi, Sicilia). Risultati di un sondaggio esplorativo. Quaderni del Museo Archeologico Regionale Antonio Salinas 8, 9–22.

[R40] Mannino MA, Catalano G, Talamo S, Mannino G, Di Salvo R, Schimmenti V, Lalueza-Fox C, Messina A, Petruso D, Caramelli D, Richards MP, Sineo L, 2012. Origin and diet of the prehistoric hunter-gatherers on the Mediterranean Island of Favignana (Egadi Islands, Sicily). PLoS One 7 (11), e49802. 10.1371/journal.pone.0049802. 23209602 PMC3509116

[R41] Mannino MA, Thomas KD, Crema ER, Leng MJ, 2014. A matter of taste? Mode and periodicity of marine mollusc exploitation on the Mediterranean island of Favignana (Egadi Islands, Italy) during its isolation in the early Holocene. Archaeofauna: Int. J. Archaeoz. 23, 133–147.

[R42] Martini F, 2007, ed. L’Italia tra 15.000 e 10.000 anni fa. Cosmopolitismo e regionalità nel Tardoglaciale. Firenze: Museo e Istituto Fiorentino di Preistoria “Paolo Graziosi”

[R43] Martini F, 2016. L’arte paleolitica e mesolitica in Italia, Millenni, 12, Firenze.

[R44] Martini F, Lo Vetro D, 2018. Grotta del Romito a Papasidero: una storia calabrese da 24.000 anni fa – Ente Parco Nazionale del Pollino, Rotonda.

[R45] Martini F, Lo Vetro D, Colonese AC, De Curtis O, Di Giuseppe Z, Locatelli E, Sala B 2007, L’Epigravettiano Finale in Sicilia. In Martini, F. (ed.), L’Italia tra 15.000 e 10.000 anni fa. Co- smopolitismo e regionalità nel Tardoglaciale. Firenze: Museo e Istituto Fiorentino di Preistoria “Paolo Graziosi”, 209–254.

[R46] Martini F, Lo Vetro D, Borrini M, Bruno S, Mallegni F, 2012a. Una nuova sepoltura dalla Grotta di Oriente (Favignana, Trapani). Scavi 2005. Atti della XLI Riunione Scientifica dell’Istituto Italiano di Preistoria e Protostoria: Dai Ciclopi agli Ecisti: società e territorio nella Sicilia preistori- ca e protostorica, 333–340.

[R47] Martini F, Lo Vetro D, Colonese AC, Cilli C, De Curtis O, Di Giuseppe Z, Giglio R, Locatelli E, Sala B, Tusa S, 2012b. Primi risultati sulle nuove ricerche stratigrafiche a Grotta d’Oriente (Favignana, Trapani). Scavi 2005. Atti della XLI Riunione Scientifica dell’Istituto Italiano di Preistoria e Protostoria: Dai Ciclopi agli Ecisti: società e territorio nella Sicilia preistorica e protostorica, 319–332.

[R48] Martini F, 2016. L’arte paleolitica e mesolitica in Italia, Millenni, 12, Firenze.

[R49] Mathieson I, Lazaridis I, Rohland N, Mallick S, Patterson N, Roodenberg SA, , 2015. Genome-wide patterns of selection in 230 ancient Eurasians. Nature 528, 499–503. 10.1038/nature16152. 26595274 PMC4918750

[R50] Mathieson I, Alpaslan-Roodenberg S, Posth C, Szécsényi-Nagy A, Rohland N, Mallick S,, 2018. The genomic history of southeastern Europe. Nature 555, 197–203. 10.1038/nature25778. 29466330 PMC6091220

[R51] Modi A, Tassi F, Susca RR, Vai S, Rizzi E, De Bellis G, , 2017. Complete mitochondrial sequences from Mesolithic Sardinia. Sci.Rep 7, 42869. 10.1038/srep42869. 28256601 PMC5335606

[R52] Olalde I, Allentoft ME, Sanchez-Quinto F, Santpere G, Chiang CWK, De Giorgio M, , 2014. Derived immune and ancestral pigmentation alleles in a 7,000-year-old Mesolithic European. Nature 507, 225–228. 10.1038/nature12960. 24463515 PMC4269527

[R53] Palma di Cesnola A, 1993. Il Paleolitico superiore in Italia. Introduzione allo studio, Firenze.

[R54] Palma di Cesnola A, 2003. Variazioni nel tempo e nello spazio dei riti funerari del Paleolitico superiore italiano, Bullettino di Paletnologia Italiana, 93–94, n.s. XI-XII (2002–2003), 1–18.

[R55] Palma di Cesnola A, 2006. L’Aurignacien et le Gravettien ancien de la grotte Paglicci au Mont Gargano. L’Anthropologie 110, 355–370.

[R56] Patterson N, Moorjani P, Luo Y, Mallick S, Rohland N, Zhan Y, Genschoreck T, Webster T, Reich D, 2012. Ancient admixture in human history. Genetics 192, 1065–1093. 10.1534/genetics.112.145037. 22960212 PMC3522152

[R57] Posth C, Renaud G, Mittnik A, Drucker DA, Rougier H, Cupillard C, , 2016. Pleistocene Mitochondrial Genomes Suggest a Single Major Dispersal of Non-Africans and a Late Glacial Population Turnover in Europe. Curr. Biol 26, 827–833. 10.1016/j.cub.2016.01.037. 26853362

[R58] Raghavan M, Skoglund P, Graf KE, Metspalu M, Albrechtsen A, Moltke I, , 2014. Upper Palaeolithic Siberian genome reveals dual ancestry of Native Americans. Nature 505, 87–91. 10.1038/nature12736. 24256729 PMC4105016

[R59] Reimer PJ, Bard E, Bayliss A, Beck JW, Blackwell PG, Bronk Ramsey C, , 2013. Int-Cal13 and Marine13 radiocarbon age calibration curves 0–50,000 Years cal. BP. Radiocarbon 55, 1869–1887.

[R60] Tomasso A, 2017. L’épigravettien: variabilité diachronique et géographique. In Olive M, ed., Campo delle Piane: un habitat de plein air épigravettien dans la Vallée du Gallero (Abruzzes, Italie centrale). Collection de l’École française de Rome. 526, 13–21.

[R61] Vigliardi A, 1996. Note sulla definizione di arte mediterranea, XIII Congr. UISPP, Colloquia 8, 97–100.

[R62] Vigliardi A, 2005. “Provincia” mediterranea e “Stile” mediterraneo: nota su un problema aperto. In Broglio A, Dalmeri G, Pitture paleolitiche nelle Prealpi venete. Grotta di Fumane e Riparo Dalmeri. Memorie del Museo civico di storia naturale di Verona, IIa Serie., Sezione Scienze dell’uomo 9, 177–178.

[R63] Villalba-Mouco V, van de Loosdrecht MS, Posth C, Mora R, Martinez-Moreno J, Rojo-Guerra M, , 2019. Survival of Late Pleistocene Hunter-Gatherer Ancestry in the Iberian Peninsula. Curr. Biol 29, 1169–1177. 10.1016/j.cub.2019.02.006. 30880015

