# Supplementary material for: Late Upper Palaeolithic hunter-gatherers in the Central Mediterranean: new archaeological and genetic data from the Late Epigravettian burial Oriente C (Favignana, Sicily)
Source: Quat Int. Author manuscript; Available in PMC 2024 Nov 21. (PMC11580030; doi:10.1016/j.quaint.2020.01.025)
Supplement: 1 [file NIHMS2034190-supplement-1.pdf]

Supplementary material

Skeletal Sex identification of Oriente C

Age at death cannot be quantified lacking suitable anatomical parts, nevertheless we observe that: exocranial sutures are not fused and there is a beginning of fusion on the endocranial aspect of the obelic suture; lt. M^3 and a fragment of upper molar (an M^1 or M^2) are unworn, six preserved long bone epiphysis (inferior right and left humerus; upper right and left radius; upper and lower left ulna) are completely fused to the diaphysis and they don't show traces of osteoarthritis. The individual probably was a young adult, maybe 25-30 year old. Oriente C lacks hip bones, hence we tried to have an indication of sex using preserved long bone midshaft and epiphysis measurements which are commonly used in sex determination in fragmentary human remains (Tab. S1).
Measurements of articulated bones, humerus and ulna, are lower than male means (z-scores -1.62 and -0.85) and close to female ones (z-scores -0.28 and 0.11); the unarticulated femur is very small, its z-score is -2.70 compared to males and -1.18 compared to females. In order to establish if such a difference between upper and lower limb bones could be found in a single individual, we computed the ratio of humeral lower epiphysis breadth (articulated bone) to femoral AP head breadth (unarticulated bone) found in the Oriente C burial and in a sample of European Upper Palaeolithic articulated individuals (Fig.S1). In Oriente C the ratio (1.393) is high, but still within the UP range (1.116-1.429) and it is intermediate between two female individuals, G.d.Enfants 3 (1.375) and Paglicci 25 (1.402). We can conclude that size proportions observed between articulated (humerus) and unarticulated (femur) bones found in Oriente C burial can be found in fully articulated UP female individuals. Hence we cannot exclude that articulated and unarticulated bones found in the Oriente C burial might belong to the same individual. In this case Oriente C would be a female as the femoral measurement is lower than the male minimum and close to the female minimum observed in our UP sample. In case the unarticulated female femur represents a different individual from articulated bones found in Oriente C burial, the sex of the latter would be uncertain, but more probably female, as far as upper limb bone measurements are concerned.

Table S1. Selected skeletal measurements of Oriente C compared to European Upper Palaeolithic male and female samples.

Sex-bone	Measure	Martin N°	n	mean	sd	min	max	Oriente C	z-score
Male humerus	Low. Epiph. Br.	M4	27	61.4	2.2	57.0	66.0	57.8	-1.62
Male ulna	Min. perimeter	M3	25	34.7	4.4	27.0	45.0	31.0	-0.85
Male femur	A-P head br.	M19	27	48.5	2.6	43.0	53.0	41.5	-2.70
Female humerus	Low. Epiph. Br.	M4	10	58.7	3.0	52.0	62.0	57.8	-0.28
Female ulna	Min. perimeter	M3	11	30.6	4.2	27.0	40.0	31.0	0.11
Female femur	S-I head height	M19	10	44.5	2.5	40.0	48.0	41.5	-1.18

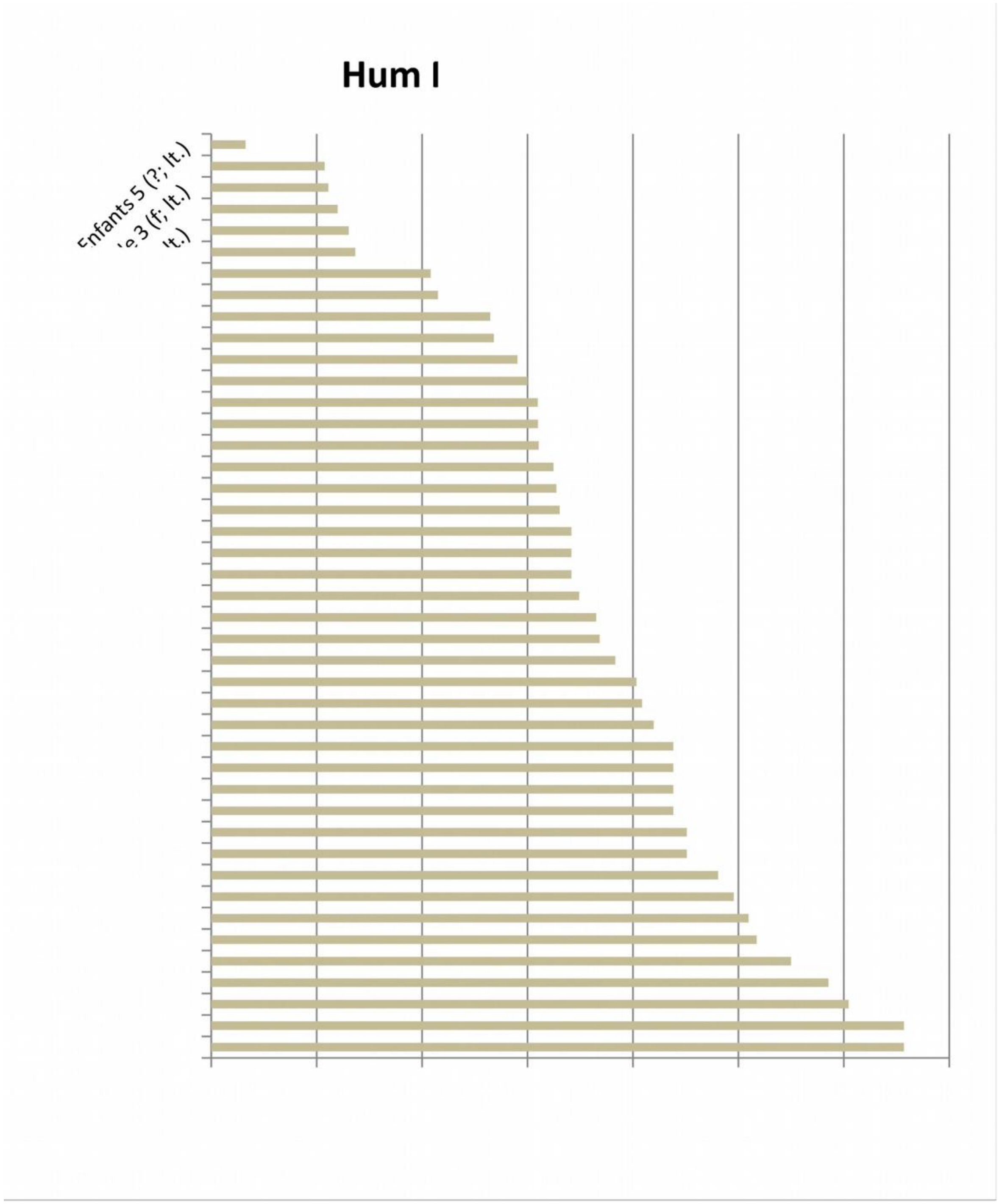

Figure S1. Humeral lower epiphysis breadth (M4) / femoral head supero-inferior height (M18) ratio in Upper Palaeolithic individuals (sex; side). Comparison sample. A.Candide: Paoli et al. (1980). Bichon:Chauvière (2008). Chancelade: Billy (1969). D.Vestonice:Trinkaus and Svoboda (2006). Ohalo:Hershkovitz et al. (1995). Ostuni (S.M. Agnano): Vacca et al. (2013). Predmost:Matiegka (1938).Sunghir: Alexeeva and Bader (2000). G. d. Enfants, Paglicci, Romito, San Teodoro and Vado all'Arancio measured by PFF.

References

Alexeeva, T.I., Bader, N.O., 2000. Homo Sungirensis: Upper Palaeolithic man: ecological and evolutionary aspects of investigation. Moscow: Scientific World.

Billy, G., 1969. Le squelette post-crânien de l'homme de Chancelade. L'Anthropologie 73, 207–246.

Chauvière, F.-X., 2008. La grotte du Bichon : un site préhistorique des montagnes neuchâteloises. Archéologie neuchâteloise 42, pp. 164.

Hershkovitz, I., Speirs, M.S., Frayer, D., Nadel, D., Wish-Baratz, S., Arensburg, B., 1995. Ohalo II H2: a 19000-year-old skeleton from a water-logged site at the Sea of Galilee, Israel. Amer. Jour. Physical Anthropology 96, 215–234.

Matiegka, J., 1938. L'homme fossile de Predmosti en Moravie (Tchécoslovaquie). II. Autres parties du squelette. Prague, Nàkladem Ceské Akademieveda Umeni.

Paoli, G., Parenti, R., Sergi, S., 1980. Gli scheletri mesolitici della caverna delle Arene Candide. Memorie dell'Istituto di Paleontologia Umana, N.S. 3, 33–154.

Trinkaus, E., Svoboda, J., 2006. Early modern human evolution in central Europe: the people of Dolni Vestonice and Pavlov. New York: Oxford University Press. pp. 489.

Vacca, E., Formicola, V., Pesce Delfino, V., Coppola, D., 2013. I resti scheletrici umani delle sepolture paleolitiche di Grotta Santa Maria d'Agnano - Ostuni (BR). In D. Coppola (a cura) "Il riparo di Agnano nel Paleolitico Superiore, la sepoltura di Ostuni 1 ed i suoi simboli" Ed. Un. di Roma Tor Vergata.
